# Two-Dimensional Patterning by a Trapping/Depletion Mechanism: The Role of TTG1 and GL3 in *Arabidopsis* Trichome Formation

**DOI:** 10.1371/journal.pbio.0060141

**Published:** 2008-06-10

**Authors:** Daniel Bouyer, Florian Geier, Friedrich Kragler, Arp Schnittger, Martina Pesch, Katja Wester, Rachappa Balkunde, Jens Timmer, Christian Fleck, Martin Hülskamp

**Affiliations:** 1 Botanical Institute III, University of Cologne, Köln, Germany; 2 Department of Biology, University of Freiburg, Freiburg, Germany; 3 Department of Biochemistry, Max F. Perutz Laboratories, University of Vienna, Vienna, Austria; 4 Max-Planck-Institut für Züchtungsforschung, Unigruppe, Köln, Germany; 5 Department of Mathematics and Physics, University of Freiburg, Freiburg, Germany; Max Planck Institute for Developmental Biology, Germany

## Abstract

Trichome patterning in *Arabidopsis* serves as a model system to study how single cells are selected within a field of initially equivalent cells. Current models explain this pattern by an activator–inhibitor feedback loop. Here, we report that also a newly discovered mechanism is involved by which patterning is governed by the removal of the trichome-promoting factor TRANSPARENT TESTA GLABRA1 (TTG1) from non-trichome cells. We demonstrate by clonal analysis and misexpression studies that *Arabidopsis* TTG1 can act non-cell-autonomously and by microinjection experiments that TTG1 protein moves between cells. While TTG1 is expressed ubiquitously, TTG1–YFP protein accumulates in trichomes and is depleted in the surrounding cells. TTG1–YFP depletion depends on GLABRA3 (GL3), suggesting that the depletion is governed by a trapping mechanism. To study the potential of the observed trapping/depletion mechanism, we formulated a mathematical model enabling us to evaluate the relevance of each parameter and to identify parameters explaining the paradoxical genetic finding that strong *ttg1* alleles are glabrous, while weak alleles exhibit trichome clusters.

## Author Summary

Trichomes, the specialized hair cells found on plant leaves, represent a model system to study how cellular interactions coordinate the development and arrangement of a collection of initially equivalent cells into regularly placed specialized cells. It was assumed that a regulatory feedback loop of positively and negatively acting factors governs these decisions. In this work, we show that trichome spacing also is controlled by the local depletion of the trichome-promoting protein TTG1. We provide evidence that binding of TTG1 to a second trichome-promoting protein, GL3, causes a depletion of TTG1 in the neighborhood of cells with elevated GL3 levels. We postulate that this leads to trichome fate determination in cells containing high GL3/TTG1 levels and prevents trichome formation in surrounding cells because of the reduced TTG1 levels. We show by theoretical modeling that this mechanism alone is capable of creating a spacing pattern and has properties that can explain even apparently paradoxical genetic observations.

## Abstract 3

Trapping of a protein in a specialized cell is shown to cause a depletion of this protein in surrounding cells, thus regulating the spacing pattern produced in plant hair cells.

## Introduction

During the development of animals and plants, specific cell types need to be placed in a regular pattern within a field of cells. In the simplest scenario, this occurs in a two-dimensional sheet of cells. Mathematical modeling of such a spacing pattern has uncovered two general principles. Both rely on the assumption that the factor promoting the formation of the specific cell type is autocatalytic. In the “activator–inhibitor” mechanism autoactivation is counteracted by the production of an inhibitor. In contrast, in the “substrate-depletion” mechanism, a substrate is consumed by the autocatalysis of the cell type promoting factor. A common requirement of both principles is significantly reduced mobility of the autocatalytic species compared to that of the inhibitor and the substrate, respectively [1].

The activator–inhibitor system is thought to generate the regular spacing pattern of leaf trichomes in *Arabidopsis* [2–4]. Trichomes are regularly distributed on the leaf surface without any reference to morphological landmarks, and clonal analysis indicated that cell lineage is not involved [5,6]. Therefore, trichomes are an ideal model system to study how single cells become regularly spaced within a sheet of equivalent cells.

Current models assume that the R2R3 MYB transcription factors GLABRA1 (GL1) and MYB23 [7–9], the bHLH factors G*LABRA3* (GL3) and ENHANCER OF GLABRA3 (EGL3) [10–12], and the WD40 repeat protein Transparent Testa Glabra1 (TTG1) [13,14] form a trichome-promoting trimeric complex due to the binding of one R2R3 MYB factor and TTG1 to a bHLH factor. Formally, this complex acts as the activator described in the theoretical models [1]. The activity of this complex is thought to be counteracted by the single R3 repeat MYB-like transcription factors TRIPTYCHON (TRY) [15], CAPRICE (CPC) [16], ENHANCER OF TRY and CPC1 (ETC1) [17], ETC2 [18], TRICHOMELESS1 [19], and CAPRICE LIKE MYB3 (CPL3) [20] through competition for binding of the R2R3 MYB factors to the bHLH protein [21]. The single R3 repeat MYB proteins are collectively considered to represent the inhibitor in the theoretical models. The active complex (AC) is postulated to activate the inhibitors, which can move into neighboring cells, where they repress the activators. This type of model is generally consistent with most data though several aspects have not been confirmed experimentally [3,4,6,22,23].

The role of *TTG1* in trichome patterning is obscure, as the glabrous phenotype of strong alleles suggests that it promotes trichome development, whereas the formation of trichome clusters in weak alleles suggests that it is involved in the inhibition of trichomes [5,24]. This dual function of TTG1 suggested to us that TTG1 has a central function in the patterning process. In this work, we identified TTG1 as the key component of a newly discovered depletion mechanism, likely to act in parallel to the above-described activator–inhibitor mechanism. We demonstrate that TTG1–YFP depletion depends on GL3, suggesting an underlying trapping mechanism, such that GL3 captures TTG1 in trichomes. Finally, we provide a mathematical model to evaluate the properties of this new GL3/TTG1 trapping/depletion mechanism.

## Results

### TTG1–YFP Protein Is Depleted in Trichome Neighboring Cells

*TTG1* is expressed in most tissues of the plant [14,25]. To determine the *TTG1* expression in young leaf parts, where trichome initiation takes place, we created transgenic plants, in which the β-glucoronidase (GUS) reporter gene was driven by a 2.2 kb promoter fragment including the 5′ UTR of *TTG1* (pTTG1:GUS). This fragment is sufficient to rescue completely the *ttg1–13* null-mutant phenotype when driving the *TTG1* cDNA (Table 1). pTTG1:GUS is ubiquitously expressed in young leaves with slightly elevated levels in incipient trichomes, and expression ceases in more mature leaf parts (Figure 1A and 1B). To determine the localization of TTG1 protein, we created a C-terminal fusion of TTG1 with yellow fluorescent protein (YFP) and an N-terminal fusion with green fluorescent proten (GFP), which both rescued all aspects of the *ttg1–13* mutant phenotype, including the seed coat mucilage, transparent testa, and trichome number when expressed under the *TTG1* promoter (unpublished data; Table 1 and Figure 2A–E). We further substantiated the functionality of this rescue construct by demonstrating that protein–protein interactions of TTG1–YFP with GL3 are indistinguishable from TTG1 in yeast two-hybrid interaction assays (unpublished data). Both fusion proteins were found in the nucleus and in the cytoplasm (Figure 2F). The integrity of the TTG1–YFP fusion protein was confirmed by western blot analysis (Figure 2G).

**Table 1:**
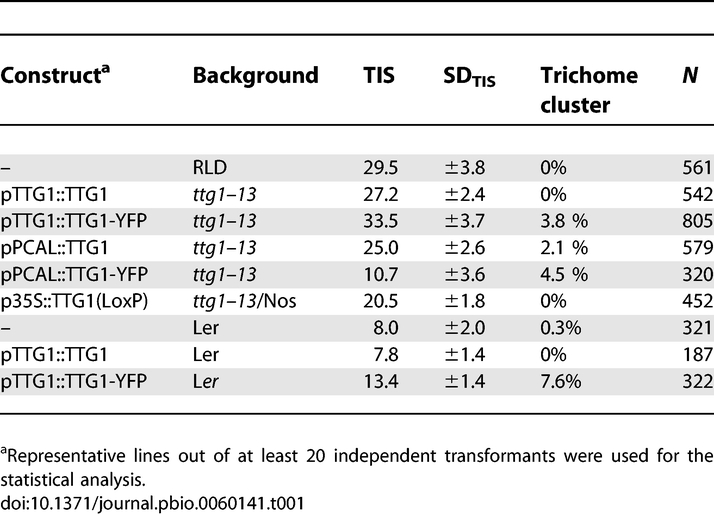
Comparison of Trichome Patterning Phenotypes

**Figure 1:**
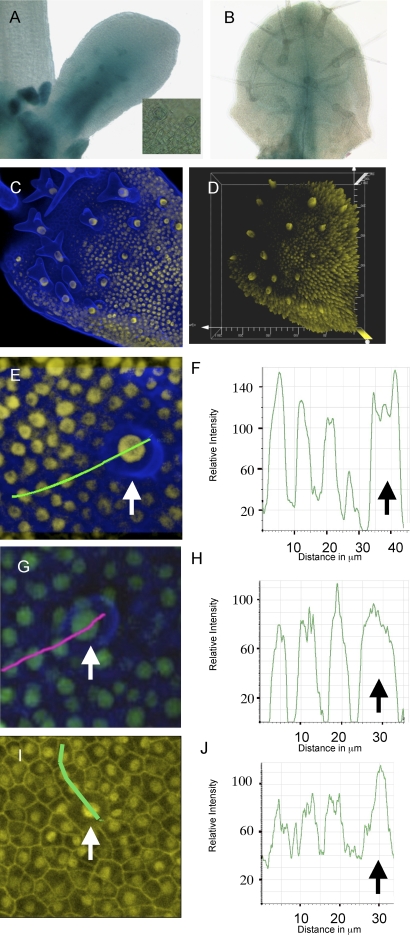
Expression and Localization Analysis of TTG1 in Developing Leaves (A) pTTG1:GUS expression in young leaf. Inset depicts a high magnification of an area with two trichomes. Note that the expression strength is similar in all cells, including trichomes. (B) pTTG1:GUS expression in mature leaf. Expression has ceased. (C) pTTG1:TTG1–YFP fluorescence in young leaf. Note that in older trichomes fluorescence is still found but that epidermal cells around them have no fluorescence. (D) Three-dimensional illustration of signal strength in (C). The fluorescence intensity is indicated by the size of the peaks. (E) pTTG1:TTG1–YFP. The arrow depicts an incipient trichome. (F) Quantification of the relative fluorescence intensity along the green line in (E). Note that the intensity drops the most in the cell next to the trichome. (G) pTTG1:GFP-NLS. The arrow depicts an incipient trichome. (H) Quantification of the relative fluorescence intensity along the pink line in (G). (I) *gl3* pTTG1:YFP. The arrow depicts an incipient trichome. (J) Quantification of the relative fluorescence intensity along the green line in (I). Yellow, YFP-specific fluorescence; blue, cell wall stained with propidium iodide (false colored).

**Figure 2:**
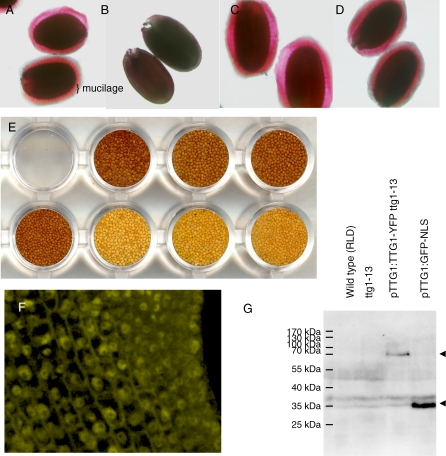
Phenotypic Description of Mutants and Transgenic Lines and Localization of TTG–YFP Fusion Protein (A–D) Seed coat mucilage stained with ruthenium red. This staining visualizes the mucilage coat as a diffusely stained zone around the seed indicated by a curly bracket. (A) wild-type ecotype RLD. (B) *ttg1–13*, no mucilage is seen. (C) *ttg1–13* pTTG1:TTG1. (D) *ttg1–13* pTTG1:TTG-YFP. (E) Seed coat color. Upper row: *ttg1–13* pTTG1:TTG1, *ttg1–13* pTTG1:TTG1-YFP line #4, *ttg1–13* pTTG1:TTG1–YFP line #1. Lower row from left to right: wild-type ecotype RLD, strong allele *ttg1–13*, weak allele *ttg1–9* and weak allele *ttg1–10*. (F) pTTG1:TTG1–YFP fluorescence. Strong fluorescence is found in the nucleus, and moderate fluorescence in the cytoplasm. This is particularly good to see in regions containing undifferentiated cells. (G) Western blot analysis to test the integrity of the TTG1–YFP and GFP–NLS fusion proteins. The TTG1–YFP fusion protein (70.5 kDa) is detected as a single band at the expected size (upper arrowhead). This band is not seen in the control lane ttg1–13, and no degradation products were found. Also the GFP–NLS fusion (31 kDa) is detected at the expected size (lower arrowhead).

The distribution of the pTTG1:TTG1–YFP fusion protein differed strikingly from pTTG1:GUS expression. Initially, in very young leaf regions, in which trichomes are not yet initiated, TTG1–YFP is detected in all cells reflecting the gene expression pattern (Figure 1C). In slightly older leaf regions, TTG1–YFP accumulates in incipient trichomes (Figure 1C, 1D, and 1E). In the cells adjacent to young trichomes, TTG1–YFP levels are the lowest, and fluorescence gradually increases with the distance from the trichome (Figure 1D and 1E). This initial observation was confirmed by quantifying the fluorescence intensity, using the Leica Confocal software (Figure 1F). On average, cells next to a trichome showed 39% of the fluorescence of that in the trichome, the cells in the second tier around a trichome 76%, and cells of the third tier 93% (*n* = 31). As a control, we measured the distribution of fluorescence of a nuclear-localized GFP under the control of the *TTG1* promoter (pTTG1:GFP-NLS, Figure 1G and 1H). The fusion to the nuclear localization signal (NLS) reduces or completely prohibits the movement of proteins [26–28], and therefore the distribution of GFP–NLS should reflect the expression pattern of the *TTG1* promoter in this assay system. Consistent with the pTTG1:GUS lines, TTG1 expression is elevated in trichome initials and ubiquitously distributed in the surrounding cells (first tier 74%, second tier 76%, third tier 77%, *n* = 30). Depletion next to the trichome cell was not found, demonstrating that the relative distribution of TTG1–YFP differs significantly from its expression pattern. Using the Mann–Whitney *U* test, the strong fluorescence reduction in the first tier is highly significant (*p* < 0.0001).

The difference between the homogeneous *TTG1* reporter expression and the non-homogeneous protein distribution could be explained in principle by two mechanisms. First, the protein stability could be controlled spatially, such that TTG1 is more stable in trichomes than in the neighboring cells. Second, the uneven distribution could result from TTG1 movement from neighboring cells into trichomes.

### Depletion of TTG1–YFP Protein in Cells Surrounding Trichomes Is Not Due to Proteasomal Degradation

To determine whether TTG1–YFP depletion around trichomes is regulated by protein degradation, we treated whole pTTG1:TTG1–YFP plants with epoxomicin, a specific and irreversible inhibitor of the proteasomal degradation machinery [29]. The TTG1–YFP protein depletion around trichomes was not affected by epoxomicin treatments, suggesting that uneven distribution of the TTG1–YFP is not caused by a difference in TTG1 stability in trichome initials and its adjacent cells (Figure 3E–G). As a control to show that TTG1 is an actual target of the 26S proteasome and that the proteasomal inhibitor was active, we used cotyledons of the same plants analyzed for the depletion of TTG1–YFP around trichomes on rosette leaves. TTG1 is expressed in cotyledons ([25], our own observation); however, TTG1–YFP protein is not detectable in cotyledons of untreated plants or control plants (Figure 3A and 3C). In plants treated with 20 μM epoxomicin for 24 h, TTG1–YFP protein could be detected in cotyledons, showing that the epoxomicin treatment was effective (Figure 3B and 3D). Control plants treated with the solvent DMSO showed no YFP-specific fluorescence in cotyledons (Figure 3C).

**Figure 3:**
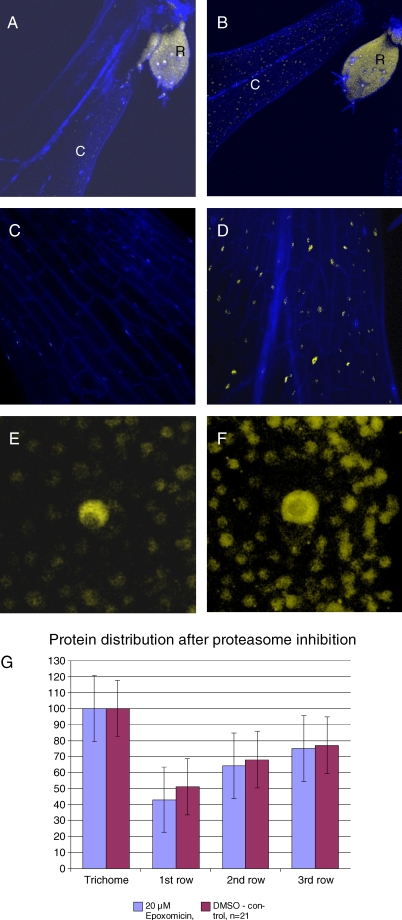
TTG1 Protein Stability (A, C, E) Control plants after 24 h of DMSO (2%) treatment. (B, D ,F) Plants after 24 h of epoxomicin (20 μM) treatment. (A and B) Rosette leaf (R) and basis of a cotyledon. Note the strong yellow fluorescence in the rosette leaf and the absence of yellow fluorescence in the cotyledon cells in the control (A) and nuclear fluorescence after epoxomicin treatment (B). (C and D) High magnification of cotyledon cells. (E and F) Depletion of TTG–YFP is seen in the control (E) as well as in epoxomicin treated plants (F). (G) Quantification of the protein distribution of control plants and epoxomicin-treated plants (*n* = 36).

### TTG1 Can Move between Cells and Acts in a Non-Cell-Autonomous Manner

The concept that TTG1 moves from neighboring cells into trichomes was proved by the following series of experiments. First, we demonstrated movement of the TTG1–YFP fusion protein from non-trichome cells into trichome cells, using the #232 activation tag line from the Poethig collection (http://enhancertraps.bio.upenn.edu/default.html, line #232). This line was identified as a line, driving the expression of the GAL4/VP16 activator, triggering expression of a *UAS* promoter driven mGFP5-ER, a GFP form localized to the endoplasmatic reticulum (ER) as a cell-autonomous marker. GFP-ER was expressed in an apparently random pattern but never in trichomes at any stage of development (Figure 4A–C). In contrast, the TTG1–YFP fusion under the control of the *UAS* promoter in this enhancer trap line showed additional YFP-specific fluorescence in initiating trichomes next to epidermal cells expressing the GAL4/VP16 activator (Figure 4A–C). This suggests that the TTG1–YFP fusion moved from the trichome neighboring cells, where it was expressed, into the trichome.

**Figure 4:**
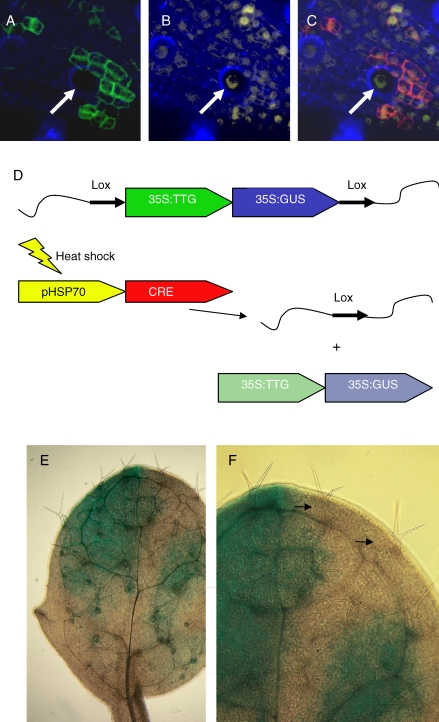
TTG1 Movement The Poethig collection GAL4/VP16 activator line #232 containing a pUAS:ER-GFP and a pUAS:TTG1-YFP construct was used to test whether TTG1 moves into trichomes. (A) GFP-specific fluorescence channel showing the expression pattern of the GAL4/VP16 driver line. Note that cells immediately next to a trichome (arrow) show strong expression (green). (B) YFP-specific fluorescence channel showing the distribution of TTG1–YFP. Note that the trichome nuclei show strong staining. (C) Overlay of (A) and (B) with the GFP shown in false color (red). (D) Outline of the Cre-Lox strategy to generate mutant *ttg1* sectors. *TTG1* and *GUS* under the control of the CaMV *35S* promoter were cloned between the lox sites, and *ttg1* mutant plants were transformed. The *ttg1* phenotype was completely rescued, and plants showed ubiquitous GUS staining (unpublished data). After saturating heat treatment, the recombination results in the deletion of the *35S:TTG1* and *35S:GUS*. All daughter cells were hence *ttg1* mutant and GUS-negative (unpublished data). (E) Cre-Lox-induced sectors. Blue regions are wild-type *TTG1* and white sectors are genetically *ttg1* mutant. Note that trichomes are also found in white sectors. (F) Higher magnification of (E) with trichomes in a white sector indicated by an arrow.

Second, we asked whether TTG1 exerts its function in a non-autonomous manner. We used the Cre-LoxP recombination system to create *ttg1* mutant sectors in plants, where wild-type-expressing cells were marked by GUS expression [30]. This was achieved by cloning the *TTG1* and the *GUS* genes, each under the control of the CaMV 35S promoter, between the two LoxP recombination sites and by introducing this construct into *ttg1–13* mutants, containing the Cre recombinase under the control of a heat-shock inducible promoter (Figure 4D). These plants showed a wild-type trichome pattern due to the rescue of *ttg1* by *35S:TTG1* (Table 1) and ubiquitous expression of *GUS*. Heat shocks were applied when the first two leaves emerged. After a saturating heat treatment of 1–2 h, no GUS staining and no trichomes were detected on leaves three and four (unpublished data). Heat-shock conditions (5–15 min) were chosen such that a recombination event excising the *35:TTG1*/*35:GUS* occurred rarely. These cells subsequently developed into large clonal sectors on leaves number three and four. As shown in Figure 4E and 4F, GUS-negative and therefore *ttg1* mutant sectors were found that clearly exhibited trichomes. This shows that TTG1 can rescue the *ttg1* mutant in a non-cell-autonomous manner.

Third, we analyzed whether TTG1 protein can actively move between cells. It has been shown that soluble GFP, 2 × GFP, and 3 × GFP (27, 54, and 81 kDa, respectively) move passively between cells with higher capacity at early stages and restricted mobility later in development [31,32]. Therefore, the size of a protein is not the main criterion for its ability to move between cells. Transport of molecules between plant cells is mainly regulated through plasmodesmata (PDs), plant-specific channels that span the cell wall and connect plant cells with each other. In recent years, several proteins have been shown to move between cells, most likely by using the PD pathway [33,34]. Hence, the potential of TTG1 to act non-cell-autonomously and to move between cells raises the question whether the 38 kDa TTG1 protein moves by actively opening the PDs. To test this general biological property of TTG1, we used microinjections in tobacco mesophyll cells (Figure 5 and Table 2). This system can be used to monitor changes in the symplasmic connectivity after injection of proteins [35]. Each set of experiments on a given leaf includes four steps. First, the injection of the small fluorescent tracer molecule acridine orange and lucifer yellow confirmed that the leaf tissue was healthy and that cells were symplasmically connected (Figure 5A). Second, 11-kDa rhodamine–dextran or 12-kDa F-dextran were injected to show that molecules larger than the plasmodesmatal size exclusion limit (SEL) for this tissue do not move into the neighboring cells (Figure 5B and Table 2) [36].

**Figure 5:**
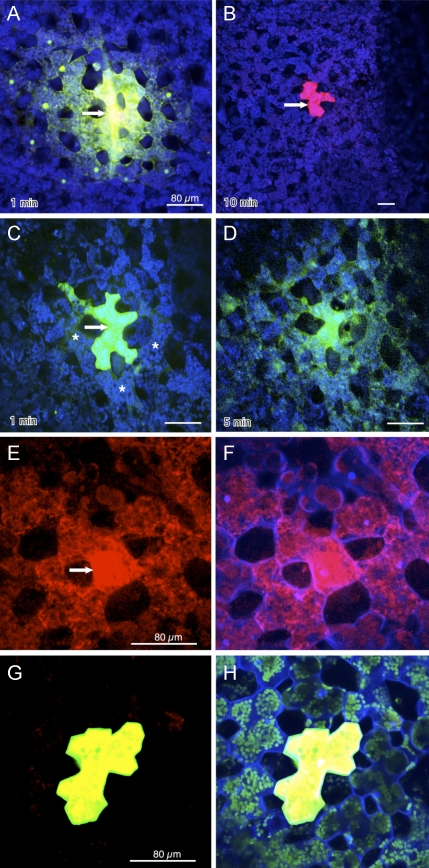
Confocal Images of Nicotiana benthamiana Mesophyll Cells Microinjected with Fluorescent Probes (A) Symplasmic connectivity is probed with the nucleic acid tracer acridine orange (red, RNA; green, DNA). After 1 min, DNA/RNA fluorescence staining is observed in nuclei of injected and neighboring cells. (B) An 11-kDa rhodamine–dextran probe remains in the injected cell (red). Image was taken 10 min after injection. **(**C) Recombinant TTG facilitates 11-kDa FITC–dextran (green) movement into neighboring cells. The fluorescent signal is detected in adjacent cells (*) after 1 min. (D) After 5 min the green fluorescent tracer moves into 3–5 cells distant from the injected cell. The blue channel shows autofluorescence of plastids (false colored). (E and F) TTG1 labeled with rhodamine (red) moves from cell to cell. (F) Merged image showing nucleic acid (nucleus) and cell wall staining with DAPI (blue). (G) GST labeled with rhodamine (red) remains in the injected cell and does not allow transport of 11-kDa FITC dextran (green). (H) Merged image with DAPI staining (blue) and autofluorescence of chloroplast (green, false colored). Arrows indicate side of injection.

**Table 2:**
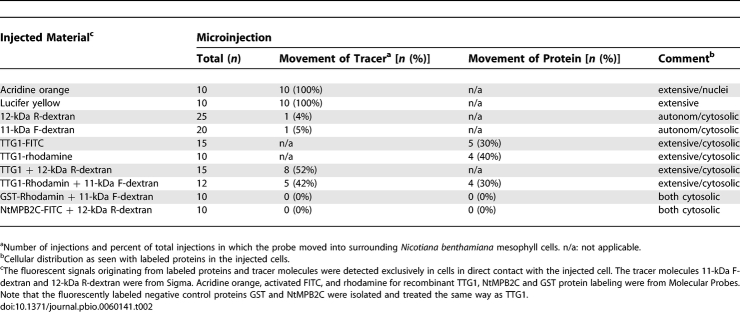
TTG1 Moves from Cell to Cell in Microinjection Assays

Third, the coinjection of the normally cell-autonomous 12-kDa F-dextran and TTG1 protein was done to test whether TTG1 can increase the SEL for this tracer. As shown in Figure 5C and 5D, the F-dextran moved out of the injected cell into neighboring cells in these coinjection experiments, suggesting that TTG1 increases the SEL. Fourth, to test directly whether the 38-kDa TTG1 protein can move, it was labeled with fluorescein isothiocyanate (FITC) or rhodamine. After injection, the fluorescent signal emitted by labeled TTG1 protein appeared within minutes in adjacent cells (Figure 5E and 5F and Table 2). GST–rhodamine and NtMPB2C–FITC were used as negative controls in these experiments [37,38]. Both proteins did not move and did not trigger movement of the tracer, indicating that the injection procedure as such did not change the movement behavior of the tracer or proteins in general. Thus, recombinant TTG1 protein shows an equivalent behavior in microinjection assays as the non-cell-autonomous KN1 protein [36]. These data indicate that TTG1 similar to KN1 increases the plasmodesmatal SEL and moves actively to neighboring cells via the intercellular transport pathway established by PDs.

Finally, we tested the movement ability of TTG1 between cell layers. For subepidermal expression studies, we used the mesophyll-specific phosphoenolpyruvate carboxylase promoter from Flaveria trinervia (ppcA1) [39]. To corroborate the specificity of the promoter in *Arabidopsis*, we used it to express a GFP–YFP fusion, which does not move between leaf tissue layers in *Arabidopsis* [40]. The GFP–YFP signal was exclusively detected in subepidermal tissue from early primordia stages on (Figure 6A and 6B). In contrast, lines expressing TTG1–YFP under the ppcA1 promoter showed additional fluorescence in the epidermal layer, showing that TTG1–YFP moved from mesophyll to epidermal tissue (Figure 6E and 6F). Consistent with this, cDNA expressed under the ppcA1 promoter rescued the *ttg1* mutant trichome phenotype equally well as under the endogenous *TTG1* promoter. Also the TTG1–YFP fusion rescued the *ttg1* mutant phenotype, though less efficiently (Table 1). In young leaves, the TTG–YFP signal was found in all epidermal cells (Figure 6E), whereas in older leaves it was found only in trichomes (Figure 6F). This finding is consistent with the earlier observation that *TTG1* is expressed only in subepidermal tissues during embryo development but is required in the protodermal tissue (the embryonic epidermis) [41]. To test whether trichomes can generally attract proteins or whether this is a specific property of TTG1, we also expressed YFP under the control of the ppcA1 promoter (Figure 6C and 6D). The YFP protein was observed in all cell layers in young tissues (Figure 6C). However, YFP did not accumulate in trichomes (Figure 6D). These data indicate that trichome-specific localization is a property of the TTG1 protein rather than due to trichome characteristics, such as a larger SEL of PDs or generally higher import rates of molecules.

**Figure 6:**
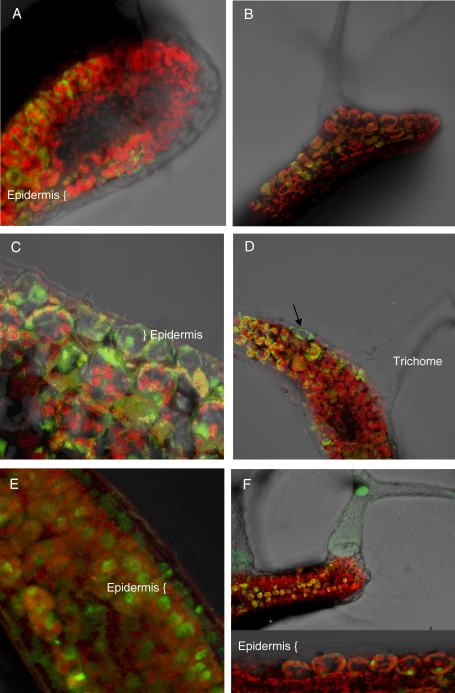
TTG1 Movement between Cell Layers (A) ppcA1:GFP–YFP in a young leaf. GFP/YFP-specific fluorescence is found in the subepidermal but not in the epidermal cell layer. (B) ppcA1:GFP–YFP in an old leaf. Fluorescence is restricted to the subepidermal cell layers. (C) ppcA1:YFP in a young leaf. YFP is found in all cells. (D) ppcA1:YFP in an old leaf. Subepidermal expressed YFP is occasionally found in the epidermis (arrow). Trichomes show little or no fluorescence as shown in this picture. (E) ppcA1:TTG1–YFP in a young leaf. Fluorescence is found in all cell layers. (F) ppcA1:TTG1–YFP in an older leaf. In the epidermis, only trichomes exhibit fluorescence. Inset shows epidermis at higher magnification. Green, specific YFP fluorescence; red, chlorophyll fluorescence.

### TTG1–YFP Depletion Depends on GL3

To understand the mechanism leading to the depletion, we tested the hypothesis that TTG1–YFP might be trapped by GL3 in trichomes. This seemed reasonable because GL3 expression is increased in trichomes relative to the surrounding cells and because GL3 strongly binds to TTG1 in yeast two-hybrid assays [12]. If the hypothesis is correct, then one would expect that TTG1–YFP would not show depletion in *gl3* mutants. As shown in Figure 1I and 1J, TTG1–YFP is ubiquitously distributed in the epidermis in plants lacking functional GL3. The quantification revealed elevated fluorescence in trichome initials and ubiquitously similar levels in the surrounding cells (first tier 79%, second tier 77%, third tier 79%, *n* = 40). These data strongly suggest that TTG1–YFP is depleted through trapping in trichome cells by GL3.

### Mathematical Modeling of the TTG1 Depletion Mechanism

We used mathematical modeling to evaluate the properties of a patterning mechanism solely based on GL3/TTG1 depletion. Therefore, we neglected the influence of additional inhibitors on the patterning mechanism. The model is based on the following assumptions: (i) TTG1 is constantly and ubiquitously expressed (shown in this work). (ii) TTG1 moves nondirectionally between cells. Although we show that TTG1 can actively open the PDs, there is no evidence for regulated transport affecting the actual rates. (iii) TTG1 forms a dimer with the GL3 protein as indicated by yeast two-hybrid results [12]. (iv) The AC enhances the expression of GL3 cooperatively. This is assumed because nonlinearity of the positive feedback is absolutely necessary for pattern formation. The data toward this end are not clear. At the whole plant level, it appears that GL3 is involved in a negative feedback loop [42]; however, at the current experimental resolution, these data do not contradict our assumption. Moreover, the GL3 homolog TT8 was shown to act in an autoactivation [43]. (v) GL3 and the AC are cell-autonomous. This assumption is based on the observation that GL3 protein does not move in the leaf (unpublished data). (vi) All components are degraded by first-order kinetics. The corresponding interaction scheme is shown in Figure 7A.

**Figure 7:**
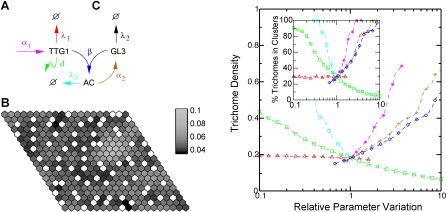
Mathematical Modeling of Trichome Patterning by Depletion of TTG1 (A) Interaction scheme. TTG1 is ubiquitously expressed at rate α_1_ (magenta arrow), degraded at rate λ_1_ (red arrow), and nondirectionally transported between cells at rate *d* (green arrow). It forms an AC with GL3 at rate β (blue arrows). The AC induces the expression of GL3 at rate α_2_ (brown arrow). GL3 and AC are degraded at rates λ_2_ and λ_3_, respectively. (B) Typical concentration pattern of total TTG1 (i.e., TTG1 + AC). Model parameters were estimated as explained in the Materials and Methods section. Light color indicates high concentration. Levels are normalized by the maximal concentration found in trichomes, which are indicated by white. A substantial amount of TTG1 is found in trichomes while it is depleted in neighboring cells. (C) Dependence of trichome density on parameters related to TTG1 function (color code as in (A); α_1_, star; α_2_, plus; β, diamond; λ_1_, triangle; λ_3_, circle; *d*, square). Parameters are changed in a range from 10% to 1000% of their estimated values. Blunt ends denote the loss of trichome patterning. Inset: Corresponding change of the percentage of trichome found in clusters.

Because the model parameters are unknown, we employed a two-step approach. First, a rescaling of model variables allowed the confinement of the parameters to relevant ranges. Second, we fitted the resulting model to the experimentally obtained relative fluorescence intensities of TTG1 in the vicinity of the trichomes. Fitting of the parameters also took into consideration the mean trichome density in the initiation zone. For parameter values and details of the optimization, see the Materials and Methods section.

A typical simulated concentration pattern of total TTG1 (i.e., TTG1 + AC) is presented in Figure 7B. The highest TTG1 levels are found in the trichomes where it is completely bound to GL3. In cells adjacent to trichomes, the level of unbound TTG1 is significantly lowered by depletion, while the level increases with distance from the trichomes.

Our rescaling and fitting procedure enabled us to estimate the model parameters and in turn to judge their relevance. We focused on the dependence of trichome density and clustering on parameters related to TTG1 function (Figure 7C). Here, trichome density is defined as the ratio of trichome cells to the total number of epidermal cells in the initiation zone of the young leaf. A decrease of the degradation rate λ_3_ of the AC (cyan line, circles) or of the transport rate *d* of TTG1 (green line, squares) results in an elevated trichome density/clustering. Conversely, an increase in the complex formation rate β (blue line, diamonds) raises the trichome density/clustering. Surprisingly, the trichome density/clustering is unaffected by a decreased degradation rate λ_1_ of TTG1 (red line, triangles). The increase of trichome density is correlated with a corresponding change of the percentage of the trichomes found in clusters (Figure 7C, inset). Note that blunt ends correspond to a loss of the trichome pattern; e.g., a decreased complex formation rate leads to glabrous plants.

These data provide for the first time an explanation for the apparently paradoxical observation that strong *ttg1* alleles are glabrous (suggesting a positive function) and weak *ttg1* alleles show clusters (suggesting an inhibitory function). While it is trivial that the absence of TTG1 in this model causes a glabrous phenotype, surprisingly, simulations of the depletion mechanism revealed that alterations of all parameters, except for the protein degradation rate, can lead to clusters.

## Discussion

In this study, we focus on the functional analysis of TTG1 in trichome patterning on *Arabidopsis* leaves. We show that *TTG1* is ubiquitously expressed with slightly higher levels in developing trichomes. The distribution of TTG1–YFP differs from the expression pattern such that the signal is strongly reduced in cells immediately next to the trichome. In showing that the proteasome inhibitor epoxomicin does not affect the protein distribution, we exclude the possibility that differential protein degradation results in the local depletion of TTG1–YFP around incipient trichomes. We demonstrate that TTG1 acts non-cell-autonomously by clonal analysis and that the TTG1–YFP protein can move within the epidermis into trichomes by using a GAL4-based expression system. Further, we show that TTG1–YFP can move between cell layers and that the TTG1 protein can open actively PDs in a heterologous system. Together these data suggest that TTG1 is redistributed from neighboring cells into the trichome by intracellular movement. What is the underlying mechanism of the observed depletion/attraction of TTG1? One possibility is that TTG1 moves freely and becomes trapped in trichomes. Alternatively, the redistribution could be achieved by directional movement into the trichomes, although both mechanisms do not necessarily rule out each other. The latter scenario is similar to that proposed for the function of auxin in the positioning of primordia in the meristematic region [44]. In this system, directional transport of auxin by the transporter PIN1 leads to an accumulation of the hormones in primordia and a reduced level of auxin in the neighborhood [44,45]. A directional transport similar to auxin is unlikely for TTG1 because TTG1–YFP can move from the cells, expressing it not only into trichomes but also into other epidermal cells (Figure 4A–C). We therefore hypothesized that TTG1 accumulates in trichomes, because it binds to another protein, as suggested for SHORT ROOT (SHR) in the root [46]. *SHR* is expressed in the stele and moves specifically into the endodermis, where it is required and sequestered in the nucleus due to interaction with SCARECROW [46]. In support of this hypothesis, we find no depletion of TTG1–YFP in *gl3* mutants, indicating that TTG1 binding to GL3 causes the depletion.

Current models explaining trichome patterning on *Arabidopsis* leaves are based on the activator–inhibitor-like mechanisms described above [2–4,47]. These mechanisms can explain the generation of a pattern in the absence of pre-existing positional information. However, not all aspects of the model have been shown experimentally. The mobility of the inhibitors was shown for CPC in the root system [48], but nothing is known about the mobility in leaves. Moreover, the theoretical requirement that the activators can autoactivate lacks experimental proof. Another problem with the current models is that various genetic data cannot be explained [3].

Our finding that in addition to the activator–inhibitor mechanism a substrate-depletion-like mechanism is operating during trichome patterning may provide some missing clues. In general, a substrate-depletion mechanism is superficially similar to the activator–inhibitor mechanism. Instead of producing an inhibitor that laterally suppresses trichome development in cells next to a developing trichome, a factor necessary for trichome development is removed from these cells. When simulating this type of mechanism, however, it turned out that the system properties are different [1,49]. In particular, it was noted that new peaks are formed at the maximum distance by the activator–inhibitor mechanism and by splitting already existing ones by the substrate-depletion mechanism [1,49].

To understand the properties of the GL3/TTG1 trapping mechanism, we formulated a mathematical model and fitted it to our experimental data to obtain a biologically relevant parameter range. This strategy enabled us to test how parameter changes affect patterning. In particular, we aimed to simulate the weak *ttg1* cluster phenotype as this genetic finding was the most confusing, because the lack of trichomes in strong *ttg1* mutants suggested that TTG1 functions as a trichome-promoting factor and the cluster phenotype in weak *ttg1* mutants pointed toward a role as a negative regulator [5,13,24,50,51]. The simulations of the GL3/TTG1 trapping mechanism revealed that changes of several parameters related to TTG1 function can result in a clustering phenotype. Thus, we can offer for the first time explanations for the apparently paradoxical genetic results on TTG1 with our new GL3/TTG1 trapping/depletion model. However, our reduced model can only partially capture the experimental observations. For example, the simulated mean trichome density as predicted by the optimal parameter set is still substantially larger than that in the wild type. We expect that more complex models involving additional patterning genes will improve the agreement between theory and experiment.

As GL3 is also a central component of all activator–inhibitor-based models, it is conceivable that the two models act in concert. We can recognize TTG1–YFP depletion at the earliest stages of morphologically recognizable trichome development. This would suggest that the trapping/depletion mechanism becomes relevant after the activator–inhibitor mechanism already has started the selection of trichomes. However, it is well possible that more sensitive microscopic techniques and more sophisticated imaging analysis tools will reveal the depletion much earlier, so we consider the relative timing of the two processes to be elusive at the moment. It will be a future challenge to combine both principles in a single model. To operate in biologically reasonable parameter ranges, it will be crucial to base such a model not only on qualitative but also on quantitative data.

## Materials and Methods

### Plant lines and growth conditions.

In this study, the wild-type ecotypes Landsberg erecta (Ler) and RLD were used. The *ttg1-1*, *-9*, *-10*, and *-13* and *gl3-1* mutant lines have been described previously [14,24,52]. The Poethig activation tag line #232 (Columbia ecotype) was a kind gift from Scott Poethig, University of Pennsylvania (http://enhancertraps.bio.upenn.edu/default.html). The heat-shock inducible *HSP:CRE*3 line containing the pCGNHCN construct in a Nossen ecotype background [30] was crossed into the *ttg1-13* mutant line (RLD background), and plants homozygous for both the transgene and the *ttg1-13* allele were isolated and crossed to TTG1-Lox lines. The TTG1-Lox construct is a descendant from the pCGNLox2a construct [30], introducing a 35S:TTG1:NOSpA cassette into the PmeI site of pCGNLox2a. The resulting plants of these crossings were used for heat-shock treatments. Plants were grown on 1 × Murashige Skoog agar (1% sucrose) plates for approximately 10 d at 20 °C under 16 h light/8 h dark conditions. Heat shock was performed by placing the plates into an illuminated incubator at 41 °C for 10–15 min. All transgenic lines were produced using the floral dip method [53].

### Constructs.

The *TTG1* promoter (position −2227 to −1 from the start codon and includes the 110 nucleotide of the 5′ UTR) was isolated from Arabidopsis thaliana ecotype Ler by PCR (forward primer, 5′-AAAGCTTAACCGAGAATGTCTCCCGACTTCTAT-3′; reverse primer, 5′-AGTCGACTCAAACTCTAAGGAGCTGCATTTG-3′) and cloned into pGEM-T vector (Promega Corporation) (pTTG-pGEM). An AscI restriction site was added by adapter ligation (5′-CTAGAATGGCGCGCCATT-3′) into the SpeI site of the vector. To generate the pTTG:GUS construct, the pTTG-pGEM was digested with AscI and SalI, and the resulting fragment was cloned into the binary gateway vector pAM-PAT-GW-GUS (GenBank accession AY02531) to replace the existing CaMV 35S promoter between the AscI and the XhoI sites.

To create the pPPCA1-pAMPAT binary vector, the 2117 bp promoter fragment of the *phosphoenolpyruvate carboxylase 1* gene (*ppcA1*) from Flaveria trinervia (GenBank accession X64143) [39] was removed from ppcA1-pBS 5′ with HindIII and religated using an oligonucleotide linker to generate an AscI restriction site. The resulting AscI–XhoI fragment was inserted into pAM-PAT-GW using the same restriction enzymes.

The yeast UAS promoter was PCR-amplified with the attachments of AscI for the forward primer and XhoI for the reverse primer. The corresponding fragment was ligated into pAMPAT-GW by exchanging the existing CaMV 35S promoter using AscI and XhoI, giving rise to pUAS-pAMPAT.

The *TTG1* cDNA (GenBank accession AT5G24520.1) was PCR-amplified with attB1 forward and attB2 reverse linker primers for Gateway BP recombination with the pDONR201 vector (Invitrogen). To create the TTG1–YFP fusion, the *TTG1* cDNA was PCR-amplified again to add a SalI site at the 5′ and a XhoI site at the 3′ of the coding sequence deleting the stop codon (forward primer, 5′-AGTCGACATGGATAATTCAGCTCCAGA-3′; reverse primer, 5′-ACTCGACAACTCTAAGGAGCTGCATTT-3′). The digested fragment was ligated into the SalI site of pUC18, then a XbaI–SacI EYFP fragment (Clontech) was fused C-terminally to *TTG1* using the same sites. The fusion was isolated using XhoI and EcoRI and ligated into pEN1a SalI–EcoRI fragment. The resulting construct was called TTG1–YFPpEN. pEYFP (Clontech) was digested with SalI and NotI and ligated into pEN1a to create EYFPpEN. The GFP–YFP fusion was constructed using an NcoI fragment of mGFP4, which was ligated in frame into the NcoI site of EYFPpEN. All constructs were sequenced. To create all of the binary constructs or yeast two-hybrid vectors, the Gateway LR Reaction System was used according to the user's manual (Invitrogen).

### Histochemical analysis and microscopy.

GUS activity was assayed as described previously [54]. After adding the X-Gluc-solution (5-bromo-4-chloro-3-indolyl-β-d-glucuronic acid), plants were vacuum-infiltrated for 15 min and then incubated at 37 °C overnight. The tissue was cleared by an ethanol series (15%, 30%, 50%, and 70% EtOH solutions at 37 °C for several hours).

Seed coat mucilage staining was done with a 0.01% ruthenium red solution for 15 min.

Light microscopy was performed using a Leica DMRE microscope using differential interference contrast optics. Images were taken using a KY-F70 3-CCD JVC camera and DISKUS software (DISKUS, Technisches Büro). Confocal laser scanning microscopy was done with a Leica TCS-SP2 confocal microscope equipped with the Leica software Lite 2.05 (LCS, Leica Microsystems). *Z*-stacks in steps of 1 or 2 μm were taken and processed using deconvolution tools of the Leica software. Quantification of fluorescence was performed using the same software. Plants were incubated for 10–15 min with a 10 μg/ml propidium iodide solution to visualize cell walls. Transverse sections were generated by embedding the tissues in 4% low-melting-point agarose and by hand sectioning using a razor blade as described by [55]*.* Images were assembled and processed using GIMP 2.2 software (http://www.gimp.org).

### Microinjections.

Recombinant TTG1 protein was produced in Escherichia coli, labeled, purified, and microinjected as previously described [38,56]. The protein concentration used for microinjection was 2 μg/μl. A Leica SP2 AOBS UV confocal microscope was employed to detect the fluorescent probes after microinjection. Tissues were scanned in sequential mode to excite and detect fluorescence probes in their specific wavelengths, and the resulting *Z*-stack (5 μm distance) images were merged using the NIH image software ImageJ (version 1.32j) (http://rsb.info.nih.gov/ij/).

### Epoxomicin treatment.

pTTG1:TTG1–YFP plants were grown on Murashige Skoog agar plates containing 1% sucrose at 22 °C for 6 d under 16 h light/8 h dark conditions and then transferred into liquid ½ MS medium containing 1% sucrose. The medium contained either 2% DMSO (control) or 20 μM epoxomicin (Sigma-Aldrich, stock solution in DMSO). The samples were vacuum-infiltrated for 15 min and incubated under the same growth conditions as previously for 24 h. After being washed with ½ MS (1% sucrose), plants were analyzed using confocal laser scanning microscopy (see above).

### Yeast two-hybrid.

Yeast two-hybrid interaction assays were performed as described previously [9]. Fusions with the GAL4 activation domain and GAL4 DNA-binding domain were performed in the pACT and pAS plasmids (Clontech).

TRY, GL3, and a truncated version of GL3 lacking 96 amino acids at the N-terminus were fused to the GAL4 activation domain in the pACT vector. TTG1 and TTG1–YFP were fused to the GAL4 DNA-binding domain of pAS. None of the constructs or empty vectors showed any self-activation in yeast.

### Western blot analysis.

Fifteen 10 d old plants (long day conditions, 24 °C) were harvested without roots, frozen in liquid nitrogen, and afterwards ground. The powder was mixed and boiled in 300 μl of sample buffer (50 mM Tris/HCl. pH 6,8, 2% (w/v) SDS, 8 M urea, 30% (v/v) glycerol, 5% (v/v) β-mercaptoethanol, and 0.5% (w/v) bromphenol blue) for 15 min followed by centrifugation (16,000*g* at 4 °C) for 15 min. Approximately 25 μl of the supernatant was analyzed by 12% SDS-PAGE, which was followed either by Coomassie staining or by western blotting and subsequent immunodetection with anti-GFP monoclonal IgG mouse antiobody (Roche). Detection was done by electrochemiluminescence.

### Mathematical model.

On the basis of the interaction diagram presented in Figure 7A, a system of coupled ordinary differential equations was derived that describes the temporal evolution of the protein concentrations of TTG1, GL3, and the AC inside each cell. The model was formulated on a two-dimensional grid of hexagonal cells with the cell index *j* = (*y*,*x*), where 1 ≤ *y* ≤ *N* and 1 ≤ *x* ≤ *M. N* and *M* denote the number of cells in the *y* and *x* directions, respectively. Periodic boundary conditions were chosen for model simulation and analysis.

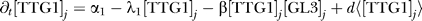


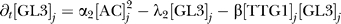


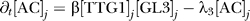



The nondirectional transport of TTG1 between cell *j* and its six neighboring cells is characterized by the coupling term

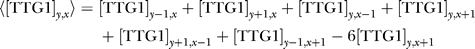



The model includes parameters α*_i_* for the expression of TTG1 and GL3 and parameters λ*_i_* for the degradation of the single proteins and the active complex. The parameter *d* is the transport rate of TTG1 between neighboring cells and the parameter β is the rate of active complex formation. To allow an assignment of reasonable parameter ranges and to reduce the number of model parameters a rescaling of the model variables was applied. All concentrations were multiplied by the factor β/λ_3_, and the new dimensionless time was expressed as τ = *t*λ_3_. The transformed, but mathematically equivalent, dimensionless equations are

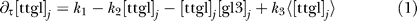


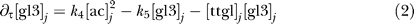


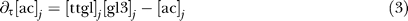



The relation between the dimensional and the dimensionless parameters *k*_1_ to *k*_5_ is given in Table 3. Let ν_0_^(*i*)^ = ([ttgl]_0_^(*i*)^,[gl3]_0_^(*i*)^,[ac]_0_^(*i*)^)^T^ denote the *i*th uniform steady state. Equations 1–3 have three uniform steady states given by

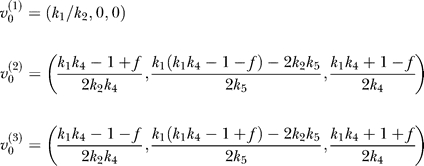
where *f* = ((*k*_1_ *k*_4_ – 1)^2^ – 4*k*_2_ *k*_4_ *k*_5_)^1/2^. For biological relevance, all three steady states must be real and positive, which restricts the range of possible values for parameters *k_i_*.

**Table 3:**
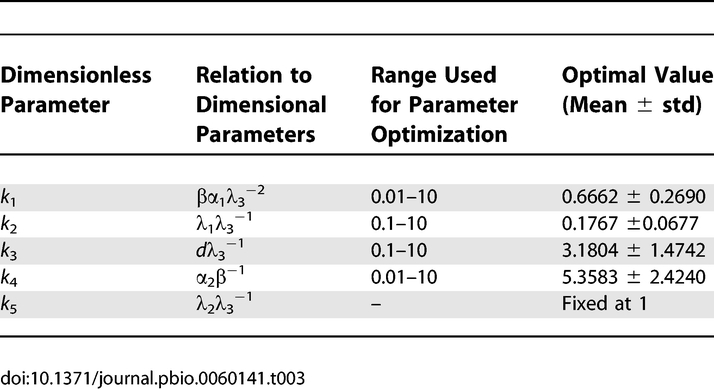
Parameters of the Mathematical Model

### Stability analysis and conditions for Turing instability.

In a pioneering work, Turing introduced the concept of pattern formation from homogeneous conditions by a diffusion driven instability; a uniform steady state that is stable for a single cell can be driven unstable by the interaction between cells [57]. On the basis of the idea of Turing, the criteria for pattern formation from a uniform steady state were derived in two steps: (i) criteria for the stability of the steady state without TTG1 mobility and (ii) criteria for an instability of the uniform steady state when adding TTG1 mobility. The stability of the steady in the absence of TTG1 mobility was analyzed by a linearization of equations 1–3 leading to ∂_τ_Δν^(*i*)^ = *J* ^(*i*)^Δν^(*i*)^ *.* Here, Δν^(*i*)^ = ν^(*i*)^ – ν_0_^(*i*)^ are small deviations from the *i*th steady state, and *J* ^(*i*)^ is the Jacobian matrix evaluated at steady state ν^(*i*)^. A steady state is stable if small deviations from it decay with time. This is the case if all eigenvalues of the Jacobian matrix have negative real parts [58]. The eigenvalues of *J* ^(*i*)^ are the roots of the characteristic equation λ^3^ + *a*_1_^(*i*)^λ^2^ + *a*_2_^(*i*)^λ + *a*_3_^(*i*)^ = 0 with the coefficients

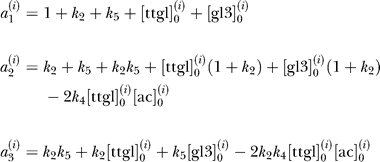



All three roots have negative real parts if the following three necessary and sufficient criteria for the coefficients of the characteristic equation are fulfilled [58]

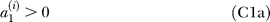


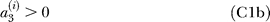


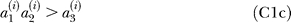



Next, we considered the stability of the steady state ν_0_^(*i*)^ including the mobility of TTG1. The temporal evolution of small spatially inhomogeneous deviations Δν*_j_* ^(*i*)^ = ν_0_^(*i*)^ – ν*_j_* from the uniform steady state ν_0_^(*i*)^ are again described by a linearization of Equations 1–3, now including the cellular coupling ∂_τ_Δν*_j_* ^(*i*)^ = *J* ^(*i*)^Δν*_j_* ^(*i*)^ + *D*〈Δν*_j_* ^(*i*)^〉. The matrix of transport coefficients is *D* and has a single entry for [ttg1] at *D*_11_ = *k*_3_. Fourier analysis was used to study the temporal evolution of spatially periodic solutions of the form Δν*_j_* ^(*i*)^ = ∑*_s_*_=1_ *^N^*∑*_r_*_=1_ *^M^*ϕ*_s_*_,*r*_^(*i*)^ e^2πi*sy*/*N*^ e^2πi*rx*/*M*^. The transformed linear equations read ∂_τ_ϕ*_s_*_,*r*_^(*i*)^ = (*J* ^(*i*)^ – 4*Dg*(*s*,*r*)) ϕ*_s_*_,*r*_^(*i*)^ with the function *g*(*s*,*r*) = sin^2^(π*s*/*N*) + sin^2^(π*r*/*M*) + sin^2^(π(*s*/*N* – *r*/*M*)). The uniform steady state ν_0_^(*i*)^ becomes unstable to small spatial variations if any of the eigenvalues of the matrix *J* ^(*i*)^ – 4*Dg*(*s*,*r*) has a positive real part. The eigenvalues of *J* ^(*i*)^ – 4*Dg*(*s*,*r*) are roots of the characteristic equation λ*_s_*_,*r*_^3^ + *b*_1_^(*i*)^(*s*,*r*)λ*_s_*_,*r*_^2^ + *b*_2_^(*i*)^(*s*,*r*)λ*_s_*_,*r*_ + *b*_3_^(*i*)^(*s*,*r*) = 0 with the coefficients: *b*_1_^(*i*)^(*s*,*r*) = (*a*_1_^(*i*)^ + 4*Dg*(*s*,*r*), *b*_2_^(*i*)^(*s*,*r*) = *a*_2_^(*i*)^ + 4*Dg*(*s*,*r*))(1 + *k*_5_ + [ttgl]_0_^(*i*)^), and *b*_3_^(*i*)^(*s*,*r*) = *a*_3_^(*i*)^ + 4*Dg*(*s*,*r*)(*k*_5_ + [ttgl]_0_^(*i*)^ – 2*k*_4_[ttgl]_0_^(*i*)^[ac]_0_^(*i*)^). If any of the three necessary and sufficient criteria

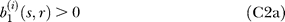


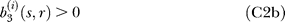


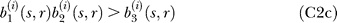
are violated, then the *i*th steady state gives rise to a Turing instability. For the analysis, we restricted all parameters *k_i_* to be real and positive. Analysis of steady state ν_0_^(1)^ revealed that conditions C1a–C1c and C2a–C2c are always fulfilled. Furthermore, if both steady states ν_0_^(2)^ and ν_0_^(3)^ are real and positive, then only ν_0_^(3)^ fulfills conditions C1a–C1c. Therefore, only steady state ν_0_^(3)^ was considered in the following. For a given parameter set, all six conditions were verified numerically. Here, it is sufficient for Turing instability if conditions C2a–C2c are violated at the maxima of *g*(*s*,*r*).

### Parameter optimization.

The parameter optimization was confined to the region in parameter space that gave rise to a Turing instability of steady state ν_0_^(3)^ as defined by the criteria given above. Additionally, parameters were restricted to the biological reasonable ranges given in Table 3. Parameters were estimated by fitting the model Equations 1–3 to the experimentally determined relative fluorescence intensities of TTG1 in the vicinity of the trichomes as well as the mean trichome density in the initiation zone of the young leaf. The optimized function was

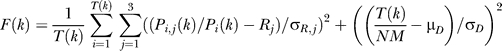
with *k* = (*k*_1_,*k*_2_,*k*_3_,*k*_4_,*k*_5_). The trichome number *T*(*k*) was determined from a numerical solution of Equations 1–3. The uniform steady state ν_0_^(3)^ plus a small inhomogeneous perturbation were used as the initial conditions. The average total [ttg1] level of the cells in tier *j* around trichome *i* is *P_i_*_,*j*_(*k*). It was normalized by the total [ttg1] level in trichome *i*; i.e., *P_i_*(*k*). *R_j_* is the experimentally determined average relative TTG1 level in tier *j*, and σ*_R_*_,*j*_ is the corresponding standard deviation. The levels are *R* = (0.387,0.765,0.935), and the standard deviation is σ*_R_* = (0.14,0.22,0.183). For the mean trichome density in the initiation zone, we used μ*_D_* = 0.075 with the corresponding standard deviation σ*_D_* = 0.035. Both values reflect the experimental observation that the mean trichome distance in the initiation zone is between 3 and 5 cells. Because the numerical solution of *T*(*k*) and *P_i_*_,*j*_(*k*) depends on the initial conditions, the optimal parameter set also depends on the initial conditions. Therefore, optimal parameters were averaged across 10 optimizations to determine the mean and standard deviation given in Table 3. For each of the 10 optimizations, a different random perturbation of the initial conditions was chosen. Parameters *k*_4_ and *k*_5_ cannot be determined simultaneously from the data. To resolve this nonidentifiability, we fixed *k*_5_ = 1. Global optimization was performed using an algorithm based on adaptive simulated annealing (Lester Ingber, http://www.ingber.com) in combination with the MATLAB interface ASAMIN by Shinichi Sakata (http://www.econ.ubc.ca/ssakata/public_html/software/). All numerical analysis was performed with MATLAB from Math Works, Inc. The predicted mean trichome density and mean percentage of the trichomes in clusters given in Figure 7C were determined from an average over 100 simulations for each parameter set.

### Accession Numbers

Accession numbers for genes mentioned in this paper from the National Center for Biotechnology Information (http://www.ncbi.nlm.nih.gov) are: pAM-PAT-GW-GUS (AY02531), *ppcA1* (X64143), and *TTG1* (AT5G24520.1).

## Abbreviations

AC: active complex
FITC: fluorescein isothiocyanate
GFP: green fluorescent proten
GL3: GLABRA3
GUS: β-glucoronidase
NLS: nuclear localization signal
PDs: plasmodesmata
SEL: size exclusion limit
TTG1: TRANSPARENT TESTA GLABRA1
YFP: yellow fluorescent protein
